# 

**Published:** 1890-11-29

**Authors:** 


### NOTICE TO CORRESPONDENTS.

All MS., Letters, Books for Review, and other matters intended for the Editor should be addressed THE EDITOR, THE LODGE, PORCHESTER SQUARE, LONDON, W.

The Editor cannot undertake to return rejected M.S., even when accompanied by stamped directed envelope.

**Notice to Advertisers and Subscribers.**

All Advertisements, Orders for Copies of THE HOSPITAL, and Business Communications should be addressed to The Publisher, *not to the Editor*, THE HOSPITAL, 140, Strand, W.C.

**Bound Volumes of "The Hospital."**

Vol. VI., containing full page portraits of T.R.H. the Prince and Princess of Wales, and Miss Florence Nightingale, is still obtainable.

Vol. VII. and all the earlier volumes are also for sale.

Orders will be received for Vol. VIII., 4s. post free. Cases for binding may be had of the Publisher, at 1s. 6d. each.

**To Residents, Secretaries, and Matrons.**

The Editor will be glad to have the Regulations and each Report as issued by Asylums, Hospitals, Convalescent and Nursing Institutions, as well as those of other Charities, and an early copy in duplicate of all Appeals and Festival Notices, etc., addressed to *The Editor, The Lodge, Porchester Square, London, W.* Any superintendent, secretary, matron, or other chief official who regularly sends such information may be registered, on application to the Publisher, to receive free of cost a cover for binding THE HOSPITAL in half-yearly volumes.

### BURDETT'S HOSPITAL ANNUAL FOR 1890

May be obtained of the Publisher, THE HOSPITAL Offices, 140, Strand, W.C., price 2s. 6d. limp boards, or 3s. 6d. cloth. All orders must be accompanied by a remittance.

### SCRAPS AND GLEANINGS.

DIPHTHERIA is decreasing at Croydon and Cheam.

THREE cases of death under chloroform have lately been recorded at St. Helens, Lancashire.

SIR MORELL MACKENZIE says it is probable he may offer himself as a candidate for Parliament.

AT Brentford a boy aged 14, has died from lock-jaw consequent on dropping a fork on his foot.

DUBLIN held a large bazaar last week in aid of the Children's Hospital in Harcourt Street.

SLEAFORD has held an enthusiastic meeting to celebrate the success of its Hospital Saturday Fund.

SIX dances are to be given at the Westminster Town Hall in aid of the Heart Hospital, Soho Square.

AT Leeds Infirmary on Saturday William Scholey died from injuries sustained in playing football.

OXFORD has refused to admit women to medical degrees by a substantial majority out of a total of 154 votes.

THE friendly societies of Bury St. Edmunds propose to celebrate their jubilee by erecting a convalescent home.

THERE were 43 applicants and only 4 vacant beds at the last election at the Royal Hospital for Incurables at Dublin.

THE Secretary of the Doncaster Infirmary and Dispensary writes to say they treated 174 in-patients last year, not 74.

MR. JOHN LONG, surgeon, of Ripley Lodge, Lower Wortley Road, Leeds, committed suicide last week by hanging himself.

INFLUENZA is prevalent in London, Edinburgh, and several provincial towns. A very bad form of typhoid is prevalent just now in London.

AXMINSTER COTTAGE HOSPITAL has a balance at the bank of £87. Captain Rogers presided at the annual meeting as Miss Conybeare was ill.

THE HON. Maud Stanley has added to her many philanthropic obligations the duties of chairman to the council of the new Hospital for Women.

PROFESSOR LISTER, the well-known surgeon, has arrived at Berlin with one of his nieces, in order that the young lady may be treated by Dr. Koch's new method.

MESSRS. BLYTON, ASTLEY, AND CO., manufacturing pharmaceutical chemists, have had their huge warehouses and laboratory at Lower Broughton destroyed by fire.

MIDDLESBOROUGH FEVER HOSPITAL seems to be inadequate; after late experiences at Birkenhead every local board should inquire into the condition of its fever hospital.

A CENSUS of persons who entered a Camberwell public-house on a recent Sunday between the hours of eight and ten p.m. showed 720 men, 152 women, and 108 couples—a total attendance of 1,118.

THE Empress Frederick has paid an hour's visit to the hospitals of Dr. Cornet and Dr. Krause, when the patients, who are being treated on Dr. Koch's new system, were presented to Her Majesty.

THE City Hospital, New York, is fitted up with telephones from each bed to the central hall; in this way friends can speak to the patients without risk of infection. English fever hospitals please note.

THE Lord Mayor, in response to the request of an influential deputation who waited upon him at the Mansion House, has consented to be the president of the Hospital Saturday Fund during his year of office.

DR. ANDERSON, professor of physiology at the University of Sydney, who is at present in England, has been requested by the New South Wales Government to proceed to Berlin to report upon Dr. Koch's method of curing tuberculosis, and to obtain a supply of lymph.

MR. CONRAD W. THIES, secretary, Royal Free Hospital, writes: My attention has been drawn to an inaccuracy in the last number of THE HOSPITAL. No beds are unoccupied at this hospital for want of funds. We are issuing a special appeal for £20,000 to enable us to complete the rebuilding of the hospital.

CITIZEN JULES GUESDE, the well-known Socialist and Revolutionary, has lost his father, who was attended on his death-bed by his daughter, a nun, who belongs to an order, the members of which devote themselves to the service of the sick. This, as the *Patrie* observes, is a better way of saving society than that of the nun's brother, Citizen Jules Guesde.

IT is announced in one of the Vienna newspapers that more than a thousand cases of influenza have occurred at Fuenfkirchen, which is a town of about 24,000 inhabitants, in the county of Baranya in Hungary. The medical officer of health for the town has sommoned a conference of all the doctors in the place in order to concert measures for combating the epidemic.

THE Rev. H. R. Haweis lectured at the Athenæum, Manchester, on cremation. He contended that the present burial system was highly injurious to the living, and that cremation presented all the advantages of burial with many advantages of its own. A specimen of cremated human remains was exhibited in the room, in a transparent casket, by members of the Manchester Cremation Society.

IN the Charity Hospital in New York a portion of a living dog's foreleg has been grafted on to a boy's leg to take the place of a bone which is wanting. The boy and the dog lie side by side in one of the hospital cots. In ten or twelve days, if the dog's limb unites with the boy's, the operation will be complete, and the last links of flesh by which the dog is connected with the boy will be cut. The dog is a black spaniel, and was encased in a plaster of Paris cast under anæsthetics.

THE Mayor of Manchester presided at the annual meeting of the Hospital for Incurables. The income from annual subscriptions had been £2,137 4s., and from other sources, £2,075 9s. 11d., making a total of £4,212 13s. 11d. The current expenditure had been £4,776 4s. 5d. The total receipts had been £6,931 9s. 10d., and the disbursements, £6,914 10s. 3d., leaving a balance in hand of £16 19s. 7d., in addition to paying off the adverse balance of last year, which amounted to £13 5s. 2d. This satisfactory result was owing mainly to the continued assistance from the Committee of the Hospital Sunday and Saturday Fund.

### The Student of Medicine.

[All communications for this Supplement should be addressed to The Editor of The Hospital, at 140, Strand, with the words, "Students' Column," written in the left hand top corner of the envelope.]

### EDITORIAL NOTES.

Students and Politics.—The students of the Edinburgh University appear to have taken a very active part in the recent election of Mr. Goschen as Rector. Our correspondent sends us an interesting account of the scenes witnessed in the Quadrangle on the day of the election. We only regret that pressure on our space prevents us from publishing it in full. There appear to have been several small scrimmages over the hoisting of the party colours in various prominent positions. The account says that, after a time a "Russellian" managed to get to the top of the statue, and fixed there an R in green and orange ribbons. The opposite party also placed the blue colours on the fountain. In the evening a great torchlight procession was held in honour of the new Rector, which started from the Old University and proceeded to the Calton Hill.

Medicine at Oxford.—The senate rejected, by a substantial majority, the new statute, introduced by Sir Henry Acland, for admitting women to study medicine in the University. Mrs. Garrett Anderson, in the *Times* the other day, replies to Mr. Case's letter which we noticed last week. The tone of her letter is very sarcastic; she writes: "Thirty years ago women might have welcomed the Oxford proposal as being a step towards getting a degree. But times have changed, and at the present day women have a wide choice of good medical qualifications, any one of which they can get on exactly the same terms as men. Why, then, should they trouble about passing examinations at Oxford, which are to lead to nothing? Will they ignore what gives them all they want for the sake of something which gives them nothing? I feel sure they will not." In another part of her letter she says: "As to the argument that if women are let off the study of Latin they will not be able to write Latin prescriptions, I think most sensible people will welcome the day when all prescriptions are written in plain English, and till it comes the amount of Latin required for prescriptions is too inconsiderable to be worth mention." With these latter remarks we cordially agree.

Oxford and Cambridge.—The old universities are naturally very slow in adopting anything new; generations of men pass away living on the traditions of those who have immediately preceded them. Oxford has, however, of late made several usually unsuccessful attempts to develope her science and medical schools, being much stimulated in this direction by the gigantic strides of Cambridge during the last few years. The science department at the latter University has carried most of the traditional obstacles before it. At the present day at Cambridge several of the ancient type of dons look on with simple bewilderment at the "dreadful" changes that have come over their university since the good old times when they first entered. Their cry is that men now come to the university to study science in preference to classics and theology.

### APPOINTMENTS.

At Charing Cross Hospital G. F. Dickinson, M.R.C.S., L.R.C.P., late house physician, has been appointed house surgeon; J. P. Harold, M.R.C.S., L.R.C.P., late demonstrator of physiology, house physician; D. J. Jones, M.R.C.S., L.R.C.P. (1st F.R.C.S.), football renown in London Welsh and C.C.H.R.F.C., resident obstetric officer. At University College Hospital, S. H. Bates, M.B., M.R.C.S., L.R.C.P., senior obstetric assistant. At the London Hospital, H. M. Speechly, M.R.C.S., L.R.C.P., house physician; F. R. Reiley, M.R.C.S., L.R.C.P., house physician; D. Brown, M.B., B.Sc., M.R.C.S., L.R.C.P., house surgeon: G. R. Killick, M.B., house surgeon. At the Royal Hospital for Diseases of the Chest, City Road, Ernest Brock reappointed resident medical officer. At the General Lying-in Hospital, Harold Burgess Osburn, M.R.C.S., L.R.C.P., house physician; at the Clinical Hospital for Women and Children, Manchester, George Harry Cooke, M.R.C.S., L.R.C.P., house surgeon; at the Western Dispensary, Rochester Row, Westminster, A. E. Cope, M.B., B.S., resident medical officer; at the Clayton Hospital and Wakefield General Dispensary, Walter Dickson, M.B., C.M., junior house surgeon; at the Glasgow Maternity Hospital, Achd. Fairlie, M.A., M.B., C.M., indoor house surgeon; at the Glamorgan and Monmouthshire Infirmary, Cardiff, Samuel Fleming, M.B., C.M., assistant house surgeon; at the Glasgow Maternity Hospital, David C. Gray, M.B., C.M., outdoor house surgeon; at the Jervis Street Hospital, Dublin, W. T. Mills, L.R.C.P.I., L.R.C.S.I., house surgeon; and at the Radcliff Infirmary, Oxford, Walter John Yurrell, B.M., B.S., house surgeon.

### STUDENTS' MEDICAL SOCIETIES.

### Guy's Hospital Pupils' Physical Society.

A meeting of this society was held on Saturday, November 15th, Mr. H. E. Durham in the chair, when Mr. T. Holmes read a paper on "Spinal Caries," of which the following is a brief abstract. The chief points dwelt upon were those relating to the symptoms, diagnosis, and treatment, (*a*) symptoms in the early cases, (*b*) symptoms in the later cases. Two typical cases of the latter were then described, one in which the lesion was in the cervical and the other in the upper dorsal regions. The analysis of five years' medical and five years' surgical cases was next given and fully discussed. Finally, some remarks were made as to the treatment, (*a*) rest, drugs, etc., (*b*) operative interference, especially as regards the treatment of pressure—symptoms by "trephining" the spine. Results in eight cases were given. Fifty-two members were present, and after the paper there was an excellent discussion, which was opened by Mr. J. B. Leathes, and continued by Messrs. Guy Mackeson, T. G. Stevens, W. K. Steele, A. W. Sheen, W. D. Loveday, A. M. Daldy, A. E. Durham, W. F. Colclough, C. H. D. Ralph, A. J. Sharp, and D. S. Long. Mr. Holmes replied in detail to various questions and criticisms of the speakers, and the meeting was closed by a vote of thanks to him for his excellent paper.

### Westminster Hospital.

The monthly meeting of the Guthrie Society was held on Thursday, 13th, at eight o'clock, Mr. A. W. Harrison in the chair. Dr. C. Mercier read a paper on "The Island of Atlantis." He referred to Plato's account of the dialogue between the Egyptian priest and Solon, where the former describes it as a vast island which was situated formerly in the centre of the Atlantic, and had subsequently become submerged. Dr. Mercier explained that it had always been a favourite theory of his that such an island had at one time existed, and gave the following reasons in support of it: (1) That in the neighbourhood of the Azores, the west coast of Ireland, and the east of Newfoundland there were extensive shallows; furthermore, that a ridge was discovered during the laying of the Atlantic cable joining them. (2) That on Easter Island there were found colossal monuments, which could not have been raised by their primitive inhabitants. (3) Most geologists were agreed that the bottom of the Atlantic had not sunk; supposing his hypothesis on the existence and disappearance of the island was correct, the sea must have risen. Now, where did all this water come from? He referred his hearers to the glacial period, at the end of the tertiary epoch, when the northern hemisphere, and most probably the southern, were to a large extent buried under enormous ice fields, that in Europe extending as far south as Saxony, these fields being often 2,000 ft. thick. If all this water were over the land in the form of ice, the sea must have been lower; when, therefore, the ice melted the low-lying Island of Atlantis was swamped by the water thus set free. (4) He considered this melting of the ice the cause of the Mosaic deluge, remarking on the similarity of the Biblical accounts with those traditions of a great deluge which exist so universally among the tribes of North America and the peoples of Polynesia. There was some discussion at the conclusion of the paper, in which Messrs. Harrison, Yearsley, and Dr. P. S. Abraham took part, the latter remarking on the support which was given to Dr. Mercier's ingenious theory by the distribution of animals.

### Edinburgh University.

The first union debate of this session was held on Wednesday November 19th in the debating hall of the union. The subject was "That Vivisection Should be Prohibited by Law." Apparently the union does not contain an anti-vivisectionist for no one came forward to support it.

### Royal Veterinary Medical Association College.

The 136th ordinary meeting of this society was held in the theatre of the College on Wednesday, November 19th, at half-past six, Mr. R. C. Tayler, vice-president in the chair. The minutes of the previous meeting having been read and confirmed, Mr. H. Sessions, F.H.A.S., read a paper on "Some Diseases of Calves." He first referred to the healthy animal, born of healthy parents, and compared this normal condition with that found in disease. Some remarks were then made upon the various modes of rearing calves, and on the cause of the "pop-bellied" condition so often met with. In his paper he strongly condemned the method of "bucket rearing," saying that sucking was the more natural and least harmful process. When the "bucket" rearing is resorted to, a separate bucket should be used for each individual with the allotted quantity of milk, and that the animals should never be allowed to drink as much as they could. Some remarks were then made upon "White Scour," as to its symptoms course, treatment, and preventive measures. Finally, "Husk" was dealt with, including its cause, symptoms, treatment, and preventive

### Student's Medical Societies—*continued.*

measures. The life history of the parasite causing the disease, was minutely considered. The paper was very well received, and gave rise to considerable discussion, in which Messrs. R. C. Tayler, Spencer, Wall, Sullivan, Manton, Harvey, Bovett, and Professor Axe took part. There were present at the meeting, the President, Professors Axe and Macqueen, Messrs. Wilde, Edwards, Walpole, and Coghill, together with 53 student members, 10 visitors and the assistant secretary.

### NOTES FROM THE SCHOOLS.

### EDINBURGH.

BOOK OF STUDENTS' SONGS.—The first meeting of the committee appointed at the Inter-University Conference to edit and publish a book of students' songs was held on November 15th. The songs to be published were finally considered and agreed to, and Messrs. Nullar Patrick (St. Andrew's) and A. Stodart Walker (Edinburgh) were appointed to see the book through the press. Professor John Stuart Blackie has agreed to write a general introduction to the book.

The managers of the Royal Infirmary have decided not to admit lady medicals to the wards of the Infirmary. At present the lady medicals of Edinburgh visit the Leith Hospital.

The election of the Students' Representative Council in the Faculty of Medicine took place on Thursday, November 20th. There are nine members of each medical year. The number of nominations were: First year, seven; second year, eleven; third, eight; and fourth, twenty-seven.

The University Musical Society has been successfully revived, and shows promise of eclipsing its former efforts. Dr. J. Greig, who is doing the work of the music chair until a successor to Sir Herbert Oakeley is appointed, has kindly taken the conductorship. The society has decided to form an orchestra, which is in a fair way of being accomplished. The Union Dramatic Society is also successful started, and intends to give an entertainment in December.

Mr. Goschen's very large majority was a surprise to all, even to his most sanguine supporters. A good deal of regret has been expressed that the contest was fought on political grounds.

### PASS LISTS.

### University of London.

The following is a list of the successful candidates at the M.B. Examination held in October last:—

FIRST DIVISION.—Francis Chas. Abbot, B.Sc., St. Thomas's Hospital; Robt. Cozens Bailey, St. Bartholomew's Hospital; Stephen Henry Bates, University College; Annette Matilda Benson, B.Sc., London School of Medicine for Women; Ellen Margaret Tinné Berthon, London School of Medicine and Royal Free Hospital; David Brown, B.Sc., London Hospital; John Henry Bryant, Guy's Hospital; William Marshall Davidson, St. George's Hospital; William McAdam Eccles, St. Bartholomew's Hospital; Percy Chas. Evans, University Edin., and Guy's Hospital; Ellen Margaret Farrer, London School of Medicine and Royal Free Hospital; John Fawcett, Guy's Hospital; Frank Grange, Charing Cross Hospital; Duncan McBean Greig, University College; Frederick William Hall, Guy's Hospital; Richard Tanner Hewlett, King's College; Aubray Dallas P. Hodges, London Hospital; Edward Victor Hugo, St. Bartholomew's Hospital; Henry Brunton Kitchin, University College; Arnold William W. Lea, Owens College and Manchester Royal Infirmary; Randle Leigh, B.Sc., University College, London, and Liverpool; James Sinclair McGowan, B.Sc., Owens College and Manchester Royal Infirmary; Alice McLaren, London School of Medicine for Women; Charles James Martin, B.Sc., St. Thomas's Hospital; Albert Edward Norburn, Guy's Hospital; T. Leighton Pennell, B.Sc., University College; Hugh James M. Playfair, King's College; Joseph Stewart Richards, Guy's Hospital; Richard Walter Richards, St. Bartholomew's Hospital; Thomas Frank Ricketts, B.Sc., and John Robertson, Guy's Hospital; James Harry Sequeira, London Hospital; Sidney Herbert Snell, and Herbert Tilley, University College; Ethel Newton Tribe, London School of Medicine for Women; Holburt Jacob Waring, B.Sc., St. Bartholomew's Hospital; Henry Woolmington Webber, Guy's Hospital; Lewis Williams, University College.

SECOND DIVISION.—Harold William Colmer Austen, St. Bartholomew's Hospital; Robert Alfred Bindley, Guy's Hospital; Herbert George Graham Cook, St. Bartholomew's Hospital; Albert Ernest Cope, College of Medicine, Newcastle-on-Tyne; Harry Corner and Bertrand Edward Dawson, B.Sc., London Hospital; Philip Rashleigh Dodwell, University College; Emily Louisa Dove, London School of Medicine for Women; George John Eady, King's College; Robert Henry Elliot, St. Bartholomew's Hospital; George Sinclair Farquharson, London Hospital; Frances Harris, London School of Medicine for Women; William Speed Hayman and Arthur Egerton Hensley, King's College; Charles Imray Kirton, London Hospital; Algernon Wilson Lyons, King's College; Minnie L. C. Madgshon, London School of Medicine and Royal Free Hospital; Joseph Moore, St. Bartolomew's Hospital; John Desmond E. Mortimer, St. Bartholomew's and Westminster Hospitals; Frederick Layton Orr, University College; Thomas George Stevens, Guy's Hospital; Helen Mary Wilson, London School of Medicine for Women; Thomas Jason Wood, University College.

**Pass Lists**—(*continued*).

**Royal College of Surgeons of England.**

The following gentleman passed the first professional examination for the diploma of Fellow at a meeting of the Board of Examiners on the 13th inst.: Herbert Edward Durham, of Cambridge University and Guy's Hospital. Eleven candidates were referred.

The following gentlemen, having passed the necessary examinations, were admitted Licentiates in Dental Surgery: George Harris Acton, Woodlands Road; James Enderby Appleton, M.R.C.S., Blackheath; William Henry Buckley, Oldham; Ernest Rogers Bull, Brecknock Road, Camden Town; Francis Burton, Norwood Road; Frederick Edward Cutts, Lancaster; William Herbert Davies, Gunnersbury; Henry Dormer, Horsley, Gloucestershire; Charles Fursman Efford, Brockley; Frederick George Hall, Caledonian Road; Walter Stanley Holford, M.R.C.S., Sutton; George Mulready Keevil, Keppel Street; William Robert Larbaleistier, Ilminster Gardens; George Lombardi, Great Marlborough Street; Cyril Darby Marson, Stafford; William May, Thorverton; Alfred Moore, Goodman's Fields; Ernest Parsons, Horseheath, Cambs.; Carl Schelling, Upper Gloucester Place; James West Summers, Norton Folgate; Louis Crowhurst Tomlyn, Florence Road; Joseph George Turner, Grange Park, Ealing; William Henry Wheatley, St. James's Road; Percy Harry White, M.R.C.S., Cadogan Square; Michael Yeatman Woolf, Marlborough Place.

The following gentlemen, having passed the necessary examinations and having conformed to the bye-laws and regulations, were, at an ordinary meeting of the council on Thursday, the 13th inst., admitted members of the College, viz.:—

F. H. Alderson, L.R.C.P. Lond., Durham University and Middlesex Hospital; R. W. Anderson, L.R.C.P. Lond., University College Hospital; H. Andrew, L.R.C.P. Lond., St. Thomas's Hospital; A. E. Ash, L.R.C.P. Lond., Manchester Royal Infirmary; W. B. Bale, L.R.C.P. Lond., Manchester Royal Infirmary; W. K. Bell, L.R.C.P. Lond., Charing Cross Hospital; G. R. Bickerstaff, L.R.C.P. Lond., St. Mary's Hospital; A. H. Bindloss, L.R.C.P. Lond., Cambridge University and St. Mary's Hospital; T. G. Brodie, L.R.C.P. Lond., King's College Hospital; F. G. Bushnell, L.R.C.P. Lond., University College Hospital; N. A. Butterfield, L.R.C.P. Lond., University College Hospital; H. A. Caley, L.R.C.P., Lond., St. Mary's Hospital; D. M'D. L. Campbell, L.R.C.P., Lond., St. Mary's Hospital; H. B. Cardew, L.R.C.P. Lond., St. Bartholomew's Hospital; A. Carney, L.R.C.P. Lond., St. Bartholomew's Hospital; H. J. Carstairs, L.R.C.P. Lond., St. Thomas's Hospital; F. J. Charlton, L.R.C.P. Lond. University College Hospital; C. Coles, L.R.C.P. Lond., St. Bartholomew's Hospital; R. C. M. Colvin-Smith, L.R.C.P. Lond., Cambridge University and St. George's Hospital; C. D. Cooper, L.R.C.P. Lond., University College Hospital; W. C. Costine, L.S.A. Lond., Liverpool Infirmary; J. M. Crocker, L.R.C.P. Lond., Yorkshire College and Leeds General Infirmary; A. Crompton, L.R.C.P. Lond., St. Bartholomew's Hospital; L. W. Dent, L.R.C.P. Lond., King's College Hospital; C. W. Emlyn, L.R.C.P. Lond., St. Bartholomew's Hospital; E. Evans, L.S.A. Lond., St. Bartholomew's Hospital; C. H. Fowler, L.R.C.P. Lond., St. Bartholomew's Hospital; B. Goddard, L.R.C.P. Lond., St. Thomas's.

### ATHLETICS.

### Football.

Association Rules.—On November 19th.

*Cambridge University* beat the *Swifts* at Cambridge, by six goals to nil. *Oxford University* were beaten by *Mr. J. H. Farmer's Eleven* at Oxford, by four goals to three. *Middlesex Hospital* drew with *Barnes*, at Barnes. The result, two goals each.

November 22nd.

*Cambridge University* beat *Chatham*, at Chatham, by three goals to nil. *Oxford University* beat *Crusaders*, at Queen's Club, West Kensington, by seven goals to four. *Guy's Hospital* beat *Barnes*, at Raynes Park, by one goal to nil. *King's College* beat *R.T.F.C.*, Cooper's Hill, at Cooper's Hill, by three goals to nil. *Philberd's Masters* beat *University College*, at Holyport, by three goals to one. *Queen's Park* beat *Edinburgh University*, at Glasgow, by six goals to nil. *St. Bartholomew's Hospital* beat *Grosvenor*, at Walthamstow, by nine goals to one. *Liverpool Regimal* beat *Medical Staff Corps* by five goals to one.

Rugby Rules—November 19th.

*Oxford* were defeated by *Cardiff*, at Oxford, by a goal to a try.

November 22nd.

*St. Thomas's Hospital v. London Scottish.*—This match was played in the Old Deer Park, Richmond, in fine weather, before a numerous company. The Scots, who kicked off and showed to the most advantage, Anderson punting into touch. Easterbrooke, after a fine pass, carried the ball into the Hospital twenty-five. The Scots played up, and a pass from Lindsay to Harvey looked like ending in a score, but the latter was just collared in time. The "Medicals'" forwards, after a fine rush, carried the ball into their opponents' quarters, but the Scotchmen, after some clever passing, transferred the game to the opposite end. After this, the ball was sent by Anderson to Lindsay, who passed to Gedge, and the latter got behind the posts, the try thus obtained was converted into a goal by Ogilvy. Thomas's played up brilliantly and Ashford got in, the place-kick failing. After change of ends, Howard obtained a try for the "Medicals," but this unfortunately was not converted, while the Scotchmen just before time obtained another try, which was converted into a goal. After a splendid game the Scotchmen were victorious by two goals and two tries to two tries. Sides:—London Scottish: K. M'Niven (back), G. T. Campbell, G. C. Lindsay, and H. T. S. Gedge (three-quarter backs), D. G. Anderson and R. F. Easterbrooke (half-backs), N. F. Henderson, J. H. Hedderwick, R. G. Macmillan, A. Methuen, T. B. Riddell, N. J. L. Ogilvy, A. Harvey, J. D. M'Donald, and R. H. M'Donald. St. Thomas' Hospital: T. O. Halliwell (back), B. J. Ogilvy, J. L. Prayne, and J. Graham (three quarter-backs), J. H.

### Athletics—*continued.*

Fisher and P. Northcote (half-backs), F. W. L. Goodline (captain), W. Jackson, C. H. Greaves, R. W. Ord, P. C. Gabbett, W. Ashford, J. P. Carver, H. Haward, and J. L. Turner.

*Cambridge University* beat *Blackheath*, on Corpus ground, at Cambridge (three goals, two tries to nil). *Lennox* beat *University College Hospital*, at Tottenham (four goals two tries to nil). *London Hospital* beat *Civil Service*, at Richmond (one goal, two tries to one goal, one try). *Richmond* beat *Oxford University*, at Oxford (one goal to two tries). *St. Bartholomew's* beat *Croydon*, at Croydon (one goal to one try). *St. Thomas's Hospital* (*Second*) beat *Middlesex Wanderers*, at Richmond (three goals, three tries to nil). *St. George's Hospital* beat *Old Devonians*, at Ealing (one try to nil). *Westminster Hospital* beat *Dental Hospital*, at Raynes Park; Westminster forwards played well together, being most of the time in their opponents' "twenty-five" (result, three goals, eight tries to nil). *Wickham Park* beat *St. Mary's Hospital*, at Wormwood Scrubbs (two goals, two tries to one try). *Edinburgh University* and *Gala* had a draw, each side scoring one goal. *Surrey Wanderers* beat *University Hospital* (three goals, two tries to nil).

### EVENTS FOR THE MONTH OF DECEMBER.

### Medical Societies.

ST. BARTHOLOMEW'S HOSPITAL ABERNETHIAN SOCIETY.—December 4th, Mr. A. Rendel, M.B., "On Renal Tumour."

ST. GEORGE'S HOSPITAL HUNTERIAN SOCIETY.—December 11th, Mr. Cotes "On Urethral Discharges."

GUY'S HOSPITAL PUPILS' PHYSICAL SOCIETY.—December 13th, Mr. A. S. Wohlmann, "On Some Recent Poisoning Cases at Guy's."

ST. MARY'S HOSPITAL MEDICAL SOCIETY.—December 3rd, "Maternity Work," Mr. H. S. Collier. Clinical Cases, Mr. P. J. Kingston. December 17th "On the Clinical Pathology of Albuminuria," Dr. R. Maguire. Clinical Cases, Mr. J. Griffith.

MIDDLESEX HOSPITAL MEDICAL SOCIETY.—December 11th, Histological subject, "Muscle and Nerve."

ST. THOMAS'S HOSPITAL MEDICAL AND PHYSICAL SOCIETY.—December 4th, Clinical and Pathological Evening. December 18th, Mr. C. R. Box, "On Experimental Pyrexia."

UNIVERSITY COLLEGE MEDICAL SOCIETY.—December 3rd, Clinical and Pathological Evening. Demonstrations; Pathological—"Diseases of Secreting Glands"; Histological—"Simple Glands."

WESTMINSTER HOSPITAL GUTHRIE SOCIETY.—December 11th.

ROYAL VETERINARY COLLEGE MEDICAL ASSOCIATION.—December 3rd, Mr. Bennett, "On Rot in Sheep"; 10th, Mr. H. H. Truman, "On Swine Fever."

### Athletics.

FOOTBALL MATCHES.

ST. BARTHOLOMEW'S HOSPITAL.—Rugby, first fifteen, December 6th, v. Tonbridge School, at Tonbridge; 13th, v. Kensington, at Wood Lane; 20th, v. North and South. Second fifteen, December 3rd, v. St. Mary's Hospital, at Wormwood Scrubs; 6th, v. Old Merchant Taylors, at Balham; 13th, v. Middlesex Hospital, at Balham.

GUY'S HOSPITAL.—Rugby, first fifteen, December 6th, v. Northampton, at Northampton; 13th, v. Old Merchant Taylors, at Willesden; 20th, v. North and South, in the South. Second fifteen, December 6th, v. Middlesex Wanderers; 13th, v. Eardley Park, at Raynes Park. Association, first eleven, December 6th, v. Chamois, at Hackbridge; 13th, v. St. Margaret's, at St. Margaret's; 20th, v. Chiswick Park, at Raynes Park.

KING'S COLLEGE.—Association, first eleven, December 6th, v. Queen's Park Rangers, at Wormwood Scrubbs; 10th, v. St. George's Hospital, at Ealing; 15th, v. Casuals, at Kensington Park. Rugby, first fifteen, December 6th, v. Catford Bridge (second), at Wormwood Scrubbs.

LONDON HOSPITAL.—Rugby, December 6th, v. Sidcup, at Sidcup; 13th, v. Brighton, at Brighton. Association, December 6th, v. Beckenham, at Beckenham; 13th, v. Salways, at Leytonstone.

ST. MARY'S HOSPITAL.—Rugby, December 3rd, v. King's College, at Wormwood Scrubbs; second fifteen v. St. Bartholomew's Hospital second, at home; 6th, v. Windsor, at Windsor; second fifteen, v. Wren and Gurneys, at home: second fifteen, 13th, v. University College School, at home. Association, December 6th, v. Polytechnic Wanderers, at Wimbledon; 17th, v. Old Westminsters, at Vincent Square.

MIDDLESEX HOSPITAL.—First fifteen, December 6th, v. Brighton, at Brighton; 10th, v. East Sheen, at Richmond; 13th, v. Old Dovorians, at Balham; 20th, v. North and South, in the South. Second fifteen, December 6th, v. St. Bartholomew's Hospital, at Balham.

ST. THOMAS'S HOSPITAL.—First fifteen, December 4th, v. Newport, at Newport; 6th, v. Swansea, at Swansea; 13th, v. Croydon, at Croydon; 20th, v. North v. South, South. Second fifteen, December 6th, v. Marlborough Nomads (second fifteen), at Lambeth; 13th, v. Wasps (second fifteen), at Lambeth.

ST. THOMAS'S HOSPITAL.—Association. December 3rd, v. R.M.C., at Woolwich; 6th, v. West Kent, at home; 10th, v. Clapham Rovers, at home; 13th, v. Surbiton Hill, at Surbiton; 20th, v. Old Etonians, at home.

UNIVERSITY COLLEGE AND HOSPITAL.—Association. First team, December 6th, v. Surbiton Hill F.C., at Surbiton; 13th, v. Civil Engineers, at Chiswick.

WESTMINSTER HOSPITAL.—Rugby. December 6th, v. Eltham House, at Raynes Park; 13th, v. Dental Hospital, at West Kilburn.

OWEN'S COLLEGE.—First team, December 4th, v. (Th.) Yorkshire College, at home; 10th, v. Salford, away. "A" team, December 6th, v. Broughton Rangers (A), at home; 13th, v. Sale (A), at home; 20th, v. Ashford House F.C., away. "B" team, December 6th, v. Blackley, away; 20th, v. Wilmslow (A), at home.

### United Hospitals Hare and Hounds.

December 6th, 13th, and 20th, ordinary runs at Raynes Park.

